# Reading the Wind: Impacts of Leader Negative Emotional Expression on Employee Silence

**DOI:** 10.3389/fpsyg.2022.762920

**Published:** 2022-06-09

**Authors:** Shu-Chen Chen, Jieqi Shao, Na-Ting Liu, Yu-Shan Du

**Affiliations:** ^1^Department of Business Administration, Ming Chuan University, Taipei, Taiwan; ^2^Heqin Honors School of Childhood Education, Ningbo Childhood Education College, Ningbo, China

**Keywords:** leader–member exchange (LMX), negative emotional expression, perceptions of psychological safety, silence, EASI

## Abstract

Employee silence has multiple negative effects on the organization. Studies on the influence of leader negative emotional expression on employee silence are extremely limited, and there are inconsistent findings for the expression of negative emotion among leaders, which highlight the need to explore boundary factors in this field. The purpose of this paper is based on EASI model to examine the impact of leaders’ negative emotional expression on employee silence through the perceptions of psychological safety. Moreover, drawing on social exchange theory, this paper proposed a moderated mediation model to explore how leader–member exchange (LMX) moderates the indirect relationship between leader negative emotional expression and employee silence through perceptions of psychological safety. We employed a bootstrapping technique to analyze the hypotheses. This study adopts two-wave surveys and the results shown that leader negative emotional expression triggered employee silence by employees’ perceptions of psychological safety. This study also demonstrated that LMX weakens the relationship between leader negative emotional expression and employees’ perceptions of psychological safety. Furthermore, LMX weakens the indirect relationship between leader negative emotional expression and employee silence through employees’ perceptions of psychological safety. Using multiphase data collection, we found that when LMX is at a low level, the indirect effect of leader negative emotional expression on employee silence through employee psychological safety is stronger. The theoretical, practical implications and future research suggestions are discussed.

## Introduction

Silence is defined as employees consciously withholding their ideas, opinions, or suggestions (Van Dyne et al., 2003; Tangirala and Ramanujam, 2008; Detert and Edmondson, 2011; Morrison et al., 2015; Wang and Jiang, 2015). Although silence is not an obvious or observable behavior, numerous employees have this response when they encounter work-related problems. A survey found that over 70% of employees don’t dare to express their opinions on work-related topics, and 85% of professionals indicated that they hadn’t presented their ideas on organization and work-related problems (Milliken et al., 2003). Several studies have demonstrated that employee silence behaviors have dysfunctional effects, including poor performance, low employee morale, low job satisfaction, and high turnover intentions (Greenberg and Edwards, 2009; Bolton et al., 2012; Knoll and Van Dick, 2013; Morrison et al., 2015). Silence behavior is generally considered widespread and harmful, but little research has been published on why and when employees choose silence (Morrison, 2014). To address this research gap, this study combines the perspectives of Emotion as Social Information Model (hereinafter referred to as EASI Model) and social exchange theory to clarify the moderating effect of LMX on the psychological mechanisms (psychological safety) triggered by the negative emotional expression of leaders.

Some research has demonstrated that employee behavior was based on leaders’ emotions (Liu et al., 2017), emphasizing that a leader’s emotions are critical in shaping employee behavior (Van Kleef et al., 2009). Employees will be affected by the leader’s facial expressions, voice expressions and other non-verbal expressions (Humphrey, 2002; Visser et al., 2013), which allows us to see that the leader emotional expression plays an important role when the leader interacts with the employees (Ashkanasy and Jordan, 2008). Drawing to the EASI (Van Kleef et al., 2009, 2010a), leaders’ emotional expression is the process of sending signals, and employees will conduct cognitive evaluation through the emotion signals conveyed by leaders (Van Kleef et al., 2010a; Melwani and Barsade, 2011). The research illustrates that employees detect situation is like reading the wind to determine whether it is safe to share their opinions with the leader (Milliken et al., 2003). Moreover, LMX is defined as “resource-based emotion communication between the leader and subordinates” (Loi et al., 2009, p. 404). Employees with high LMX need not judge carefully whether a situation is harmful when talking to a leader (Liu et al., 2017). Conversely, employees with low LMX must detect situations sensitively on the basis of emotional information displayed by a leader. This mentality may be the result of low degrees of emotional attachment, trust, and support from leaders (Uhl-Bien and Maslyn, 2003; Cropanzano and Mitchell, 2005). Integrating EASI Model (Van Kleef et al., 2010a) and social exchange theory (Graen and Uhl-Bien, 1995), we constructed a moderated mediation model, to examine the relationship between leader negative emotional expression and employees’ psychological safety is moderated by LMX and whether this relationship subsequently affects employee silence through psychological safety indirectly.

This study fills some gaps in the research and make several contributions. First, in the research on leaders’ negative emotional expression, most research has focused on the influence of leaders’ negative emotional expression on voice. For example, the study by Chi et al. (2018) investigated the impact of leader negative emotional expression on employee upward voice, while the study by Song et al. (2019) also took the influence of leaders’ negative emotional expression on subordinates’ voice as a research topic. In view of this, we found that the research on the effect of leader negative emotional expression on employee silence is extremely limited. Some researchers have shown that the negative emotional expression of the leader will reduce the employee voice (e.g., Milliken et al., 2003; Chi et al., 2018; Song et al., 2019). However, voice and silence are two separate and different concepts. Generally, voice leads to positive workplace outcomes, while silence has a detrimental impact on organizational development (Morrison, 2014). When employees engage in silence behavior, the management of the organization lacks specific and critical information from front-line employees, making it impossible for managers to identify and correct problems (Milliken and Morrison, 2003; Tangirala and Ramanujam, 2008). Many companies experience such trouble or even go out of business because of employees’ silence behavior (Xu et al., 2015). Thus, we emphasize the need to explore the impact of leader negative emotional expression on employee silence.

Second, few studies have explored psychological safety as a mediator in research on the negative emotional expression of leaders. The study by Liu et al. (2017) used the EASI model to illustrate the influence of leaders’ affective state on employee voice through psychological safety, but it focused more on the impact of the employee’s assessment of the leader’s emotion on voice through psychological safety. By comparison, we used a more direct variable (i.e., leader negative emotional expression) and identified leaders’ emotional expression as social information that affects employees’ psychological safety based on the EASI model to explain the relationship between leader negative emotional expression and employee psychological safety.

Finally, it is obvious that leaders’ emotions affect employees (Eberly and Fong, 2013). Among existing studies, the findings related to leaders’ negative emotional expression are inconsistent (Chi and Ho, 2014). Some studies have suggested that leaders’ negative emotional expression has a negative impact on leadership effectiveness (Game, 2008; Connelly and Ruark, 2010; Schaubroeck and Shao, 2012). Other researchers have proposed that leaders’ negative emotional expression is positively correlated with performance and employee effort levels (Sy et al., 2005; Visser et al., 2013). Negative emotions are inevitable in organizations, so it is necessary to further explore the impact of leaders’ negative emotional expression on employees (Lindebaum and Fielden, 2010), in particular the boundary factors that affect leaders’ emotional expression. In the research model developed by Chi and Ho (2014), personal factors (e.g., follower conscientiousness) and social factors (e.g., perceived leader power) are incorporated into the model as moderators to explore the impact of leader negative emotional expression on employee performance. The study by Xu et al. (2015) investigated the impact of abusive supervision on employee silence and incorporated LMX as a moderator into the research model. Although similar studies have examined LMX as a moderator, we have not found LMX to be tested as a moderator in a study investigating the impact of leader negative emotional expression on employee silence. To fill these gaps, our study investigated the moderating role of LMX, refining the influence process by providing relational context about the negative effects of leader negative emotional expression. Most existing leadership research has focused on the leader-based or relationship-based domains (Graen and Uhl-Bien, 1995; Lian et al., 2012), and research on the interaction with psychological safety is limited (Newman et al., 2017). Our study considered leader negative emotional expression and LMX at the same time and examined their interactional impact on employees from the perspective of social exchange theory, which contributes to the existing literature on leadership and psychological safety.

**FIGURE 1:**
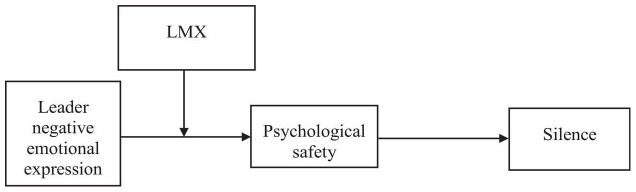
Theoretical model.

This study has several contributions. First, although silence behavior is generally considered harmful for individuals and organizations, few studies have begun to notice the silence behavior that depresses existing opinions and suggestions (Van Dyne et al., 2003; Tangirala and Ramanujam, 2008; Greenberg and Edwards, 2009; Wang and Jiang, 2015). This study explores the impact of leaders’ negative emotional expression on employee silence which enriches the literature on silence and leadership. Second, drawing on EASI, this study contributes to the extension of existing leadership and emotion research by identifying leader emotional expression as a social information to affect employee’s psychological safety (Van Kleef et al., 2009). Furthermore, this study combined the perspectives of EASI and social exchange theory to clarify the moderating effect of LMX on the psychological mechanisms (psychological safety) triggered by the negative emotional expression of leaders. A moderated mediation model of employee silence was proposed. Figure 1 demonstrates the proposed theoretical model in this study.

## Literature Review and Hypothesis Development

### Silence

Silence refers to employees already have opinions but suppress potentially important ideas or concerns about work-related issues consciously (Wang and Jiang, 2015). The construct related to silence is voice, which is defined as the act of presenting suggestions and opinions (Tangirala and Ramanujam, 2012). When employees provide suggestions and opinions rarely, it is regarded as low voice, but low voice and silence are different constructs. When addressing voice behavior, leaders usually record the number of suggestions and opinions employees present during a period of time (e.g., Detert and Burris, 2007; Tangirala and Ramanujam, 2012). A low voice does not necessarily indicate that employees are consciously silent. A low voice may indicate that the employee does not have any ideas or suggestions to share with others, whereas silence behavior indicates that the employee has ideas and opinions but withholds them consciously. Current research on employee silence (e.g., Tangirala and Ramanujam, 2008; Detert and Edmondson, 2011; Wang and Jiang, 2015) has specifically addressed whether employees consciously hide information.

Several studies have shown that employee silence is a passive reaction that may be harmful for organizations (Bolton et al., 2012; Morrison, 2014). Organizations cannot rectify potential problems and obtain ideas for continual improvement without accurate and timely information from employees. The lack of information can cause severe dysfunction (Tangirala and Ramanujam, 2008). Employee silence causes unfavorable organizational consequences, including poor performance, low employee morale, and decreased organizational performance (Greenberg and Edwards, 2009).

### Leader Negative Emotional Expression and Employee Silence: Psychological Safety as a Mediator

Few studies have focused specifically on why employees remain silent regarding potentially critical problems and concerns. The key finding of these studies was that silence stems from fears regarding the risks of speaking up (Milliken et al., 2003; Detert and Edmondson, 2011). When employees have ideas, they evaluate their social situation first, and then decide whether to express their opinions or remain silent (Liu et al., 2015). Leader negative emotional expression is a kind of observable presentation of leaders’ negative emotion. When interacting with employees, leaders can express negative emotion through verbal or non-verbal forms, which will then affect employee behaviors (Visser et al., 2013). EASI model argued that emotions are social information and have a social function (Van Kleef et al., 2009, 2010a). Followers use leaders’ emotional expressions to infer information about feelings and attitudes. EASI model combines individual and social factors in the leader negative emotional expression-follower silence relationship. Leaders’ emotional expressions convey verbal and non-verbal signals (Verbeke, 1997) that affect employees’ perceptions and reactions (Goleman et al., 2001). To perform well in social activities, employees must pay more attention to the leader’s emotional expressions (Van Kleef et al., 2010a; Melwani and Barsade, 2011).

Drawing on EASI, leader negative emotional expression can be regarded as a social information for employees, and employees may evaluate the leader’s emotional expression through leader’s tone, facial expressions and gestures (Gooty et al., 2010). When the leader expresses negative emotion, the employee infers it is a negative feedback that the leader is dissatisfied with the employee’s performance, and these social cues affect employees’ attitudes and behaviors (Van Kleef et al., 2009). Studies have demonstrated that leaders’ negative emotions play a central role in employees’ willingness to express their opinions (Liu et al., 2017). If the employee observes leader expressing negative emotion, they may regard this social information as leader unsatisfied with them and would be afraid of the negative social cues (Chi and Ho, 2014). Psychological safety reflects the degree to which employees fear negative results when they express their ideas (Liang et al., 2012). The social information of leader negative emotional expression may reduce the employee’s psychological safety (Liu et al., 2017), and the decrease of psychological safety may fuel employees to hide existing ideas consciously (Van Dyne et al., 2003; Cheng et al., 2014; Edmondson and Lei, 2014). Therefore, we propose:

*Hypothesis 1: Psychological safety mediates the relationship between leader negative emotional expression and employee silence.*

### The Moderating Role of Leader–Member Exchange

Social exchange theory (Blau, 1964) posits that social exchange is a type of relationship-oriented perception when an individual interacts with others. LMX pertains to the long-term quality of the mutual relationship. Employees with high LMX tend to believe that their workplace interactions with the leader are mutually beneficial, trusting, and safe (e.g., Dulebohn et al., 2012), and they need not judge carefully whether a situation is harmful when they are talking to the leader (Liu et al., 2017). Therefore, the decline in the psychological safety of employees with high LMX may be mitigated when faced with leader negative emotional expression. Conversely, employees with low LMX have a low degree of emotional attachment, trust, and support for their leader (Uhl-Bien and Maslyn, 2003; Cropanzano and Mitchell, 2005). When a leader expresses negative emotions, they are sensitive to whether the situation is harmful based on the emotional information displayed by the leader. The employees use the leaders’ emotional cues to evaluate whether to expresses their opinions or be silent (Liu et al., 2017). The inconsistent information between leader negative emotional expression and LMX results in an uncontrollable and unpredictable working environment (Greenbaum et al., 2012), which reduces employees’ psychological safety (Beehr et al., 2003). Therefore, we propose:

*Hypothesis 2: LMX weakens the relationship between leader negative emotional expression and psychological safety.*

### Integrated Moderated Mediation Model

According to the EASI Model, leader negative emotional expression is the process of sending signals. If employees observe that the leader is expressing negative emotion, they may regard this as social information that the leader is dissatisfied with them, which can cause fear of the negative results. Therefore, the negative emotional expression of a leader may reduce employees’ psychological safety (Liu et al., 2017). However, this relationship is moderated by LMX. When LMX is high, employees naturally feel psychologically safe and are less likely to be sensitive to whether a situation is harmful to themselves. Conversely, employees with low LMX must be sensitive to leader negative emotional expression to determine whether a situation is unfavorable to them and may worry about the negative consequences caused by expressing opinions. The reduction of psychological safety caused by leader negative emotional expression may force employees to reduce risks to themselves, consciously triggering silence behaviors (Van Dyne et al., 2003; Tangirala and Ramanujam, 2008). Therefore, we propose:

*Hypothesis 3: LMX moderates the indirect effect of leader negative emotional expression on employee silence through employee psychological safety, and the indirect effect is stronger when LMX is lower.*

## Materials and Methods

### Sample and Procedure

Before the survey began, one the authors contacted HR managers and asked if they could help gather data from their companies of employment. We received affirmative responses from the HR managers of different companies. Via communication with the HR managers of these companies, we identified 286 respondents who would be willing to participate in the survey. This convenience sampling method of collecting data from HR managers has been widely used by researchers (Bavik et al., 2017). We explained to the participants the purpose and steps of the survey, and at the beginning of each questionnaire we ensured that the survey was voluntary and anonymous. Items in the questionnaires were originally written in English and the back-translation approach was used (cf. Brislin, 1986) to translate them into Chinese. According to the suggestions of prior researchers (Podsakoff et al., 2003, 2012), the survey was separated into two phases at an interval of 4 weeks. Specifically, participants provided their rating of their leaders’ negative emotional expression and their own psychological safety at Time 1. After 4 weeks (Time 2), participants evaluated LMX and their silence behavior. We gave a gift worth $3 to everyone who participated in the survey.

The research analysis included four stages. First, we tested all constructs’ component reliability (CR), average variance extracted (AVE), convergent validity, and discriminant validity (Carrasco and Jover, 2003). Second, the distinctiveness of the assessed variables was estimated. We adopted the suggestion from Anderson and Gerbing (1988) and used Mplus software (Muthen and Muthen, 2012) for CFA [CFA, robust maximum likelihood method (MLM) estimator]. Third, we employed the PROCESS macro for SPSS (Hayes, 2013) to conduct the regression analysis to test the hypotheses. Bootstrapping was used to examine the indirect and moderating effects (i.e., H1 and H2). Finally, to examine H3, we conducted the bootstrapping technique to examine the significance of the moderated mediation effect (Edwards and Lambert, 2007; Preacher et al., 2007).

Data were collected from several companies at multiple time points to avoid common method variance (CMV) (Podsakoff et al., 2003). At Time 1, 286 questionnaires were distributed and 277 valid responses were retrieved. At Time 2, we received 212 valid responses, and the overall response rate was 74.13%. The average age of respondents was 38.81 years, and 67.9% were college graduates. The industries of participants were services (37.3%), manufacturing (16%), finance (14.2%), government (5.7%), and others (26.8%).

### Measures

#### Leader Negative Emotional Expression

Leader negative emotional expression was assessed using the negative expressivity section of a seven-item scale developed by Gross and John (1995). Employees were asked to provide ratings on a five-point Likert scale (1 = *strongly disagree*, 5 = *strongly agree*) by using their immediate leaders as references. “Whenever my leader feels negative emotions, I can easily see exactly what they are feeling” is a sample question. Cronbach’s alpha of this scale was 0.92.

#### Psychological Safety

Psychological safety was measured using five items from the scale by Liang et al. (2012). Participants rated the items from 1 (*strongly disagree*) to 5 (*strongly agree*). “In my work unit, expressing my true feelings is welcomed” is a sample question. Cronbach’s alpha of this scale was 0.93.

#### Leader–Member Exchange

O’Donnell et al. (2012) reported that the exchange relationship between leaders and subordinates is rarely the same, and leaders treat each subordinate slightly differently. We measured this variable using employee self-reports based on previous methods (i.e., Xu et al., 2012; Brees et al., 2014; Peng et al., 2014; Peng and Lin, 2016). Graen and Uhl-Bien’s (1995) seven-item scale was employed to evaluate the daily work relationship between leaders and employees. All items were rated on five-point Likert scales from 1 (*strongly disagree*) to 5 (*strongly agree*). “I have a good working relationship with my leader” is a sample question. Cronbach’s alpha of this scale was 0.92.

#### Silence

Silence was measured using Tangirala and Ramanujam’s (2008) five-item scale. Participants were asked to rate the extent to which they withheld ideas, concerns, or information regarding critical work-related problems from 1 (*strongly disagree*) to 5 (*strongly agree*). “Although I had ideas for improving work in my [work group], I did not speak up” is a sample question. Cronbach’s alpha of this scale was 0.90.

#### Control Variables

Employees’ gender, age, position level, and education level were controlled, which is consistent with studies on silence behavior (e.g., Tangirala and Ramanujam, 2008; Wu and Hu, 2009). Male and staff were encoded as 0, female and manager were encoded as 1.

## Results

Table 1 presents the correlations. Cronbach’s α values of the variables exceeded 0.7 for all constructs (leader negative emotional expression = 0.88, psychological safety = 0.92, LMX = 0.89, employee silence = 0.88) (Gerbing and Anderson, 1988). The average variance extracted (AVE) of all constructs exceeded 0.5. We used Fornell and Larcker’s (1981) approach to examine the discriminant validity of the constructs, and we determined that the square roots of the AVEs were greater than the correlations for all pairs of constructs (Table 1). Therefore, the relevant constructs demonstrated discriminant validity. Furthermore, the fit of the hypothesized four-factor model (leader negative emotional expression, psychological safety, LMX, employee silence; χ^2^/*df* = 1.74; GFI = 0.89; CFI = 0.93; RMSEA = 0.07) was superior to that of the one-factor model (χ^2^/*df* = 5.2; GFI = 0.71; CFI = 0.84; RMSEA = 0.17), two-factor model (χ^2^/*df* = 4.02; GFI = 0.57; CFI = 0.66; RMSEA = 0.14), and three-factor model (χ^2^/*df* = 2.49; GFI = 0.71; CFI = 0.84; RMSEA = 0.10). These findings demonstrated that the four-factor model exhibited the optimal fit.

**TABLE 1:** Means, standard deviations, and correlations.

Variables	Mean	SD	1	2	3	4	5	6	7	8
1. Gender^a^	0.51	0.50	—							
2. Age	38.81	11.20	–0.07	—						
3. Position level^b^	0.21	0.41	–0.20**	0.23**	—					
4. Education level ^c^	1.86	0.55	0.13	–0.20**	0.15*	—				
5. Leader negative emotional expression	3.15	1.00	–0.08	0.07	0.06	−0.05	(0.59)			
6. Psychological safety	3.73	0.90	0.06	–0.24**	–0.02	–0.02	−0.40**	(0.75)		
7. LMX	3.77	0.73	0.06	–0.06	0.06	−0.03	–0.01	0.41**	(0.67)	
8. Employee silence	2.59	0.77	–0.11	–0.06	–0.16*	–0.28**	0.08	−0.13	−0.17*	(0.61)

*p < 0.05; **p < 0.01 (two-tailed). ^a^ 0 = male, 1 = female. ^b^ 0 = staff, 1 = manager. ^c^ 1 = junior high school and below, 2 = senior or professional high school/bachelor’s degree, 3 = graduate degree. Square roots of AVEs are in brackets.

### Hypothesis Testing

To test our hypotheses, we used the regression-based PROCESS macro for SPSS (Hayes, 2013). During the mediating test, a significant negative relationship was found between leader negative emotional expression and psychological safety (β = −0.35, *p* < 0.001), and a significant negative relationship was found between psychological safety and employee silence (β = −0.14, *p* < 0.05; Table 2). Additionally, the indirect effect of leader negative emotional expression on employee silence through psychological safety was significant (indirect effect = 0.05, CI_95%_ = [0.00, 0.11] excludes zero). The bootstrapped 95% confidence interval of the indirect effect via psychological safety did not include zero (0.00, 0.11), indicating that psychological safety mediated the relationship between leader negative emotional expression and employee silence (Shrout and Bolger, 2002). H1 was thus supported.

**TABLE 2:** Results of regression analysis and moderated-mediation effect.

	Psychological safety				Employee silence
	Model 1	Model 2			Model 3
Constant	5.56***(0.40)	6.05***(0.70)			4.62***(0.50)
Gender ^a^	0.08 (0.11)	0.03 (0.10)			–0.14 (0.10)
Age	−0.02*** (0.01)	−0.02 (0.00)			−0.01 (0.00)
Position level^b^	0.20 (0.15)	0.10 (0.13)			−0.20 (0.13)
Education level ^c^	−0.17 (0.11)	−0.13 (0.09)			−0.39***(0.10)
Leader negative emotional expression (LNEE)	–0.35***(0.06)	–1.13***(0.20)			0.01 (0.06)
LMX		–0.13 (0.17)			
Psychological safety					–0.14*(0.06)
LNEE × LMX		0.21*** (0.05)			
R^2^	0.22	0.42			0.13
F	11.47***	20.93***			5.30***
	Effect	Boot SE	Boot LLCI	Boot ULCI	
Indirect effect	0.05	0.03	0.0021	0.1112	
Moderated-mediation effect (LMX)					
Low	0.07	0.04	0.0044	0.1567	
Medium	0.05	0.03	0.0013	0.1086	
High	0.03	0.02	0.0014	0.0772	
	Index	Boot SE	Boot LLCI	Boot ULCI	
Index of moderated-mediation	–0.03	0.02	–0.0741	–0.0020	

*p < 0.05; ***p < 0.001 (two-tailed). ^a^ 0 = male, 1 = female. ^b^ 0 = staff, 1 = manager. ^c^ 1 = junior high school and below, 2 = senior or professional high school/bachelor’s degree, 3 = graduate degree.

**FIGURE 2:**
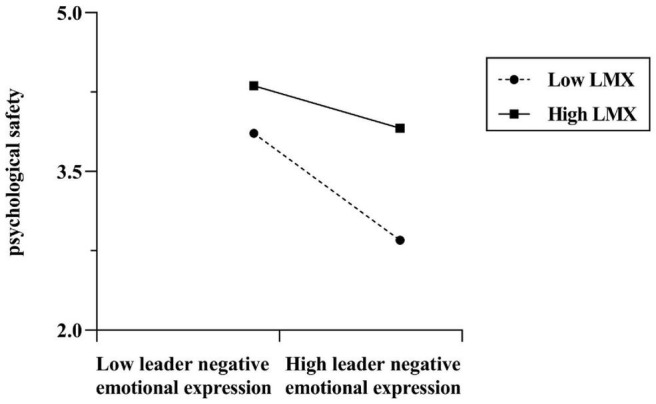
Moderating effect of LMX on the relationship between leader negative emotional expression and psychological safety.

We employed the moderated mediation analysis, using Preacher et al. (2007) bootstrapping process, to test H2. As illustrated in Table 2, the interaction of leader negative emotional expression and LMX on psychological safety was significant (β = 0.21, *p* < 0.001), which illustrated that LMX moderated the association between leader negative emotional expression and psychological safety. The line chart for high (M + 1SD) and low (M – 1SD) LMX is presented in Figure 2. When LMX was high, the negative relationship between leader negative emotional expression and psychological safety was weaker than that when LMX was low. Therefore, H2 was supported.

We used a bootstrapping approach (Hayes, 2013) with resampling and 95% confidence intervals to examine the significance of the moderated mediation effect. The results are presented in Table 2. When LMX was higher, the indirect effect was weaker (indirect effect = 0.03, CI_95%_ = [0.00, 0.08] excludes zero) than when LMX was low (indirect effect = 0.07, CI_95%_ = [0.00, 0.16] excludes zero) or medium (indirect effect = 0.05, CI_95%_ = [0.00, 0.11] excludes zero). The index of moderated mediation revealed statistical differences (indirect effect = –0.03, CI_95%_ = [–0.07, –0.00] excludes zero). Therefore, H3 was supported.

## Discussion

Silence is an act whereby employees conceal potentially valuable thoughts or suspicions concerning work-related topics. This silence can be harmful and threaten the overall development of an organization (Morrison, 2014). Studies have recommended research on the effect of leaders’ emotions on employee behavior (Morrison, 2011; Liu et al., 2017). By integrating EASI (Van Kleef et al., 2010a) with the literature on silence, we investigated the role of leader negative emotional expressions in employees’ silence. The conceptualization and examination of silence in our research involved dynamic interaction between leaders and employees. Employees who intentionally suppressed critical communication with leaders’ experience psychological problems (Morrison and Milliken, 2000). Moreover, our research results are consistent with empirical evidence on “reading the wind” (Dutton et al., 1997), indicating that leader negative emotional expression had a passive effect on psychological safety based on signal pathways, which then affected employee silence. This study demonstrates that psychological safety mediates the negative effect of leader negative emotional expression on employee silence. Our research adds to the leadership literature by demonstrating that leaders’ emotional expression critically influences employees’ perceptions and behaviors (Gooty et al., 2010), and enhances understanding of the effect of leaders’ negative emotional expression on employee silence through psychological safety.

Research on the EASI Model has indicated that the social functions of emotions are affected by boundary factors, including personal traits (e.g., agreeableness) (Van Kleef et al., 2010b) and relevant characteristics (e.g., power differences) (Van Kleef et al., 2004). Our study extends the EASI model and LMX literature by theorizing the moderating role of LMX in attenuating the effects of leaders’ negative emotional expression. This study explored how LMX affects employees’ perception of psychological safety when leaders’ emotional expression sends signals and indirectly affects employee silence behaviors through psychological safety. The results found that the psychological safety of employees is less affected by leaders displaying negative emotions when they have higher LMX levels, subsequently, they are less likely to choose silence. On the contrary, the psychological safety of employees with low LMX have a greater impact. The reduction of psychological safety caused by leader negative emotional expression may force employees to reduce risks to themselves, consciously triggering silence behaviors (Van Dyne et al., 2003; Tangirala and Ramanujam, 2008). This research enhances our understanding that in the workplace, LMX quality plays an important role in influencing people’s psychological safety during encountering leader emotion expression.

This study makes three key contributions. First, we contribute to the growing body of research on leader negative emotional expression by examining its effects on employee silence. Our study responds to the call by Tepper et al. (2007) for researchers to conduct further research on employees’ passive behavior, rather than focusing only on obvious and easily observed aggressive reactions. When employees face negative emotions from their leaders, some employees react aggressively, which can exacerbate or even end the relationship with the leader (Tepper et al., 2007). Others choose to respond passively. Silence is a threatening passive response that should be studied due to its detrimental effects on organizational development (Morrison, 2014). Our findings extend this research stream by showing that being silent in the workplace is a passive response to confront leaders that is used by employees who perceive negative emotional expressions from leaders.

Second, the results related to the moderating effects of LMX add new insights to the existing leadership literature to clarify the boundary conditions of the relationship between leader negative emotional expression and psychological safety. Our findings suggest that, compared with employees with high LMX, the psychological safety of low-LMX employees declines more rapidly in the face of negative emotional expression from their leaders. That is, when a leader’s negative emotional expression occurs in the low-quality leader–member relationship, it has a greater negative impact on the employee’s psychological safety. Using social exchange theory, we link leader negative emotional expression and LMX to important employee-level outcomes (i.e., employee silence). At the same time, we incorporate psychological safety as a mediator into the investigated research model, which helps to improve our knowledge and understanding of the complex nomological network embedded in psychological safety (Newman et al., 2017).

Finally, our findings extend the emerging but limited literature on precursors of silence (Morrison, 2014). Specifically, our findings illustrate the key role of leaders’ negative emotional expression in the process of generating employee silence, especially in the context of low LMX. The mediating role of psychological safety further explains that employees with low psychological safety are more likely to behave silently because they are afraid of the negative consequences of speaking out. Therefore, this study not only investigated the influence of leadership on employee silence behavior choice, but also confirmed an underlying moderating mechanism. Our findings further confirm previous findings that when employees are confronted with negative emotional expression from their leaders, employees’ voice behavior decreases (Milliken et al., 2003; Chi et al., 2018; Song et al., 2019).

### Practical Implications

These findings have several practical implications. First, a leaders’ emotion is a tool that can be effective if controlled appropriately but is often ignored in this field (Huy, 2002). Organizations need to focus on inhibiting leaders’ negative emotional expression, as this can have costly consequences. Organizations are responsible to make leaders clear about the terrible consequences of negative emotional expression, especially for those employees with low LMX who are more likely to remain silent. Organizations should formulate appropriate training programs to teach management to control negative emotions toward employees, thereby reducing the frequency of leader negative emotional expression (Robinson and O’Leary-Kelly, 1998). Moreover, the potential role of psychological safety requires organizations to pay more attention to employee psychological health status. Organizations can provide some support as a way to alleviate the situation that employees’ lower psychological safety in the workplace. For example, organizations can provide some psychological counseling services on a regular basis to understand the psychological health status of employees and the reasons for the decline of employee psychological safety. In addition, employees with low LMX are more affected and consider more about “reading the wind” (Dutton et al., 1997). Leaders must pay particular attention to negative expressions of their emotions when communicating with employees with low LMX. For instance, leaders with a short tenure who have not developed high LMX with their employees particularly need to be informed of the results of this study (Bauer and Green, 1996) because receiving employees’ constructive ideas and suggestions and achieving environmental innovation is beneficial (Sauer, 2011). Finally, leaders should adjust their negative emotional expression depending on the situation to prevent employee psychological safety decline and silence. For example, leaders should not express negative emotions to employees without reason. Instead, leaders should inform employees of the reasons for their anger to limit the effects on psychological safety.

### Limitations and Future Research Directions

This study has several limitations. Silence behavior is an implicit behavior that is not easily observed by others. Therefore, we used the approach presented by Xu et al. (2015) to measure this variable through employee self-reports. The data were obtained from the same source, which may have caused CMV. We applied researchers’ suggestions (Podsakoff et al., 2003, 2012) and performed a two-wave survey separated by 1 month to reduce CMV. Moreover, Podsakoff et al. (2012) concluded that a significant interaction effect in the model is strong evidence that data do not have CMV. The interaction results in this study were significant, and the indirect effects of different conditions of the moderating variable were different, indicating that the CMV effect was weak.

Future studies can explore other personal or organizational contextual factors that affect the negative relationship between leader negative emotional expression and the psychological safety of employees, such as whether the organizational climate is one in which ideas and suggestions are generally valued (Ekvall, 1996). In addition, the sample comprised full-time workers in Taiwanese companies, which generally have high power distance (Aryee et al., 2007). Employees may have a high tolerance for leaders’ negative emotional expression. Therefore, cross-cultural studies can be performed to determine the differences between Eastern and Western cultures and understand the effect of different cultures on employees’ experiences with leader negative emotional expression.

## Data Availability Statement

The raw data supporting the conclusions of this article will be made available by the authors, without undue reservation.

## Author Contributions

S-CC acted as the Principal Investigator and oversaw the study in its inception to completion. JS was responsible for data collection, writing the manuscript, and conceptualizing the models. N-TL contributed to rewriting of the manuscript in subsequent drafts after the initial submission. Y-SD was responsible for data collection. All authors contributed to the article and approved the submitted version.

## Conflict of Interest

The authors declare that the research was conducted in the absence of any commercial or financial relationships that could be construed as a potential conflict of interest.

## Publisher’s Note

All claims expressed in this article are solely those of the authors and do not necessarily represent those of their affiliated organizations, or those of the publisher, the editors and the reviewers. Any product that may be evaluated in this article, or claim that may be made by its manufacturer, is not guaranteed or endorsed by the publisher.
